# Diagnostic quality of steady state free precession imaging of cardiac valve morphology in pediatric/congenital heart disease

**DOI:** 10.1186/1532-429X-11-S1-P115

**Published:** 2009-01-28

**Authors:** Oscar J Benavidez, Ashwin Prakash, Kimberlee Gauvreau, Andrew J Powell, Tal Geva

**Affiliations:** Childrens Hospital Boston/Harvard Medical School, Boston, MA USA

**Keywords:** Aortic Valve, Left Ventricular Outflow Tract, Valve Annuli, Valve Morphology, Mitral Valve Annuli

## Objective

To evaluate cine Cardiac Magnetic Resonance – Steady State Free Precession (CMR-SSFP) imaging quality and diagnostic accuracy in the assessment of cardiac valve morphology.

## Background

Evaluation of cardiac valve morphology has not been considered an indication for cardiac MRI due to suboptimal imaging quality; few studies have examined this objectively. MRI techniques utilizing CMR-SSFP may have overcome this limitation. Evaluation of CMR-SSFP diagnostic imaging quality in the assessment of valve morphology has not been performed for pediatric/congenital heart disease. Comparison of CMR-SSFP diagnostic accuracy of aortic valve morphology to echocardiography has not been previously performed.

## Methods

**Figure 1:**
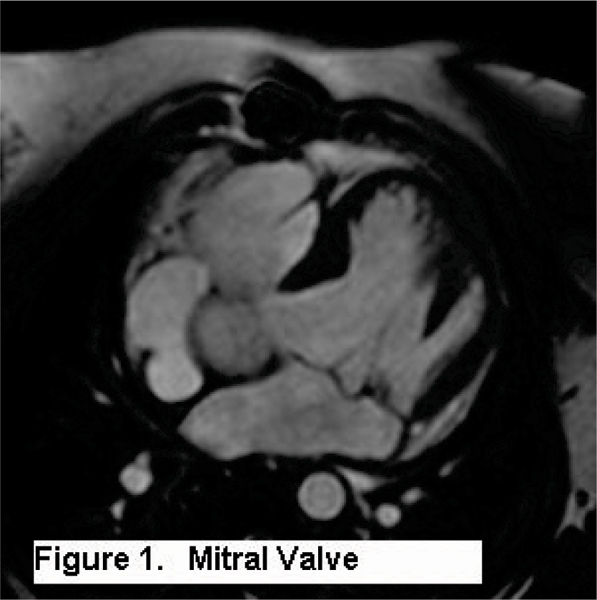
**Mitral valve**.

We retrospectively reviewed 234 consecutive pediatric/congenital cardiac MRI cases. A diagnostic clarity score (Table 1) was assigned to the tricuspid, mitral valve annulus, leaflets, chordae and papillary muscles. High-quality diagnostic imaging is defined as a clarity score of 1 or 2. The clarity score of the mitral (Figure 1) and tricuspid valves was assessed by examination of standard cine CMR-SSFP imaging in two-chamber, four-chamber and short-axis views. Clarity score of the aortic valve annulus and leaflets was assessed when long axis imaging of the left ventricular outflow tract and cross-sectional imaging of the aortic root were performed. Among patients with aortic valve imaging, we compared morphologic diagnosis by CMR-SSFP to echocardiography.

**Table 1:** Diagnostic clarity score

1	No blurring, excellent diagnostic data
2	Mild blurring, very good diagnostic data
3	Moderate blurring, diagnosis possible
4	Severe blurring, diagnosis uncertain
5	Non-diagnostic, diagnosis not possible

## Results

**Figure 2:**
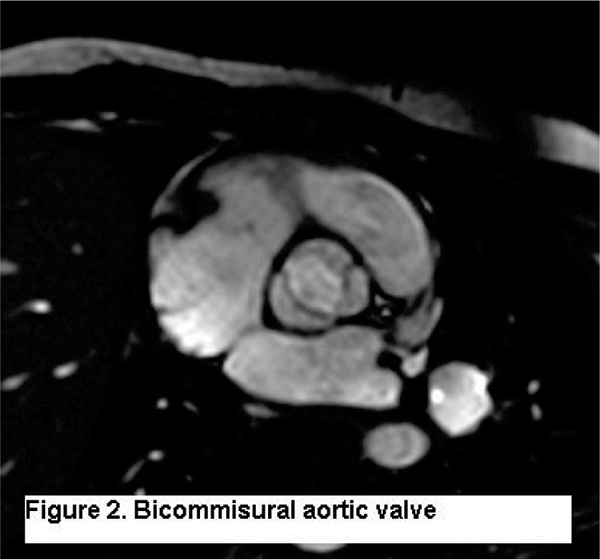
**Biocommisural aortic valve**.

Patient age ranged from 1 month to 65.1 years (average 17.2 years). Table 2 illustrates the proportion of valve components with a high diagnostic clarity scores. There was no difference in weight or body surface area between those with an average mitral or tricuspid clarity score of < 2 (excellent or very good clarity) vs. ≥ 2 (fair to poor clarity). Among the 39 cases with aortic valve specific imaging, 27 (69%) had an echocardiogram for comparison. CMR-SSFP correctly identified the aortic valve morphology in all 27 cases. CMR-SSFP correctly identified the affected commissure in 13 of 13 bicommissural aortic valves (Figure 2) and correctly identified 14 of 14 tricommissural aortic valves.

**Table 2:** Proportion of valve components with a high-quality diagnostic imaging (Diagnostic clarity scores 1 or 2)

	Annulus	Leaflets	Chordae	Papillary muscles
Tricuspid Valve	80%	64%	30%	53%
Mitral Valve	74%	69%	36%	77%
Aortic Valve	72%	80%	-	-

## Conclusion

In the majority of cases CMR-SSFP produces high diagnostic quality imaging of cardiac valve morphology in congenital/pediatric cardiac MRI. The valve components with the highest diagnostic clarity score are tricuspid and mitral valve annuli, leaflets and papillary muscles and aortic valve annuli and leaflets. CMR-SSFP however produced high clarity images of chordae in only a minority of cases. Aortic valve morphology can be diagnosed with a high degree of reliability.

